# Influence of Single Deuterium Replacement on Frequency of Hydrogen Bond Dissociation in IFNA17 under the Highest Critical Energy Range

**DOI:** 10.3390/ijms232415487

**Published:** 2022-12-07

**Authors:** Alexander Basov, Mikhail Drobotenko, Alexander Svidlov, Maria Bezhenar, Eugeny Gerasimenko, Arkadii Moiseev, Vadim Malyshko, Anna Dorohova, Andrey Drozdov, Mikhail Baryshev, Stepan Dzhimak

**Affiliations:** 1 Department of Fundamental and Clinical Biochemistry, Kuban State Medical University, 350063 Krasnodar, Russia; 2 Department of Radiophysics and Nanotechnology, Kuban State University, 350040 Krasnodar, Russia; 3 Laboratory of Problems of Stable Isotope Spreading in Living Systems, Federal Research Center the Southern Scientific Center of the Russian Academy of Sciences, 344006 Rostov-on-Don, Russia; 4 Department of Technology of Fats, Cosmetics, Commodity Science, Processes and Devices, Kuban State Technological University, 350072 Krasnodar, Russia; 5 Scientific Department, Kuban State Agrarian University, 350004 Krasnodar, Russia; 6 Laboratory of Environmental Mass Spectrometry, Institute of Analytical Instrumentation of the Russian Academy of Sciences, 198095 Saint-Petersburg, Russia

**Keywords:** open states, rotational movements of nitrogenous bases, deuterium, DNA, dynamics of a double-stranded DNA molecule

## Abstract

The effect of single substitutions of protium for deuterium in hydrogen bonds between pairs of nitrogenous bases on the open states occurrence probability at high critical breaking energies of these bonds has been studied. The study was carried out using numerical methods based on the angular mathematical model of DNA. The IFNA17 gene was divided into three approximately equal parts. A comparison of the open states occurrence probability in these parts of the gene was done. To improve the accuracy of the results, a special data processing algorithm was developed. The developed methods have shown their suitability for taking into account the occurrence of open states in the entire range of high critical energies. It has been established that single ^2^H/^1^H substitutions in certain nitrogenous bases can be a mechanism for maintaining the vital activity of IFNA17 under critical conditions. In general, the developed method of the mathematical modeling provide unprecedented insight into the DNA behavior under the highest critical energy range, which greatly expands scientific understanding of nucleobases interaction.

## 1. Introduction

Experimental study of the DNA structure is difficult because of several problems; the most important of which is the limitation of the spatial resolution of available research tools [1,2]. Despite the development of methods for studying single molecules, they have a few limitations for studying the mechanics of DNA [3,4]. For this reason, mathematical modeling is one of the main modern methods for studying DNA molecular dynamics, its mechanical movements, open states, denaturation bubbles [5,6,7]. This method, despite several simplifications, allows us to consider various aspects of DNA functioning with great accuracy [8,9,10]. In the article [11], using a combination of atomic force microscopy (AFM) and modeling of atomic molecular dynamics (MD), an attempt was made to experimentally observe the dynamics of open states. The experiment was carried out on short ring DNA, while many microphotographs were taken, after which, using a custom Python script, the images were corrected for further morphological analysis. As a result, denaturation bubbles were recorded, which, in turn, leads to DNA compaction. This indicates the importance of combining the experimental and mathematical modeling methods, which allows us to obtain much more information about the dynamics and open states of the DNA molecule [12].

Earlier, we found that the ingress of a deuterium atom into hydrogen bonds between pairs of nitrogenous bases increases the probability of occurrence of open states by 0.22–0.60% [13]. In addition, it has been shown that the probability of the bubbles formation (length from 12 to 27 nucleotides) depends on the localization of the deuterium atom in the DNA molecule and may differ significantly from the probability of the occurrence of open states in general [14]. The participation of deuterium atoms in the formation of hydrogen bonds in the DNA molecule can cause a change in the time of transmission of genetic information, thus, even a slight change in the isotopic state of the medium can affect changes in metabolic processes in living systems [15,16,17]. It is known that non-radioactive isotopes of biogenic elements (^2^H/^1^H, ^13^C/^12^C, ^15^N/^14^N, ^18^O/^17^O/^16^O) have a significant effect on the rate of biochemical reactions, physiological processes, growth, and development of unicellular and multicellular living organisms with different levels of organization of energy metabolism and metabolic rate [18,19,20,21,22,23,24,25].

The aim of this work is to study the effect of a single deuterium substitution at the frequency of hydrogen bond dissociation in IFNA17 in the range of the highest critical energies, based on the mechanical model of DNA [26], without simplifications and averaging [27,28].

## 2. Results

**Figure 1:**
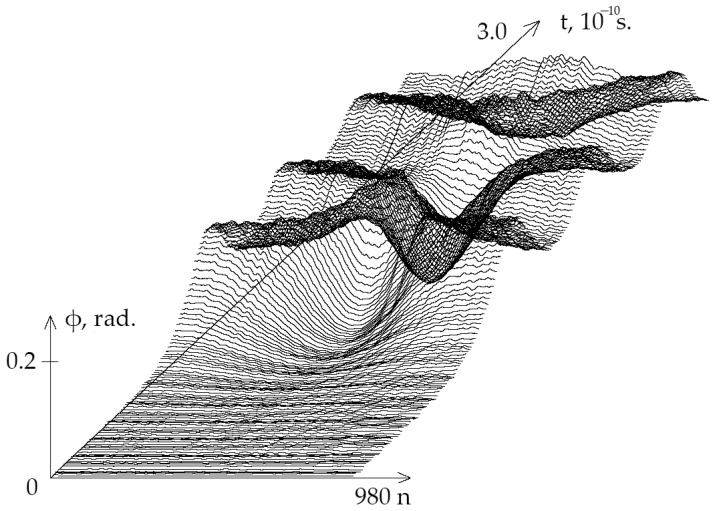
Graphs of angular deviations of the 1st chain of DNA molecule nitrogenous bases in the gene encoding interferon alpha 17 under $E_{cr}^{H}$ equals $0.581\times 10^{-22}$ N·m over period [$0,t=3.0\times 10^{-10}$ s.].

On Figure 1 the graphs of the angular deviations of the 1-st chain of DNA molecule nitrogenous bases under the first $E_{cr}^{H}$, which equals $0.581\times 10^{-22}$ N·m over period from 0 to $3.0\times 10^{-10}$ s are presented.

**Figure 2:**
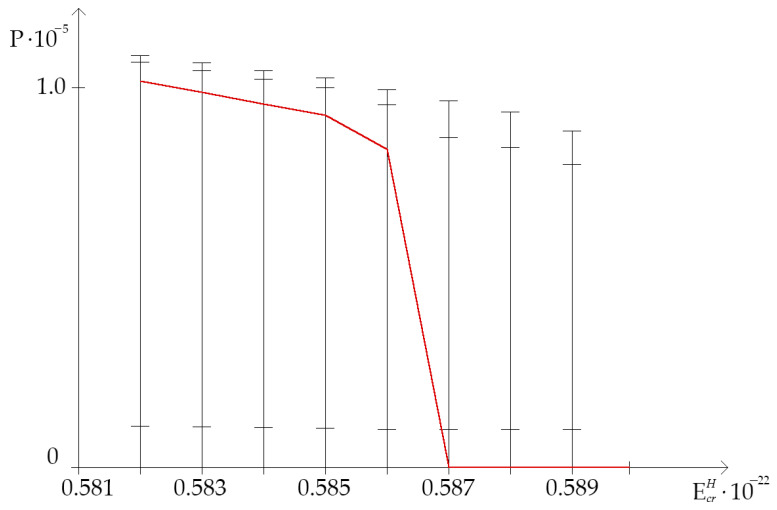
Dynamics of the open state (OS) occurrence in gene encoding interferon alpha 17 dependent on the H-bond dissociation energy under natural condition and after the single ^2^H/^1^H replacement (with gradation of OS occurrence frequency by modified Basov–Jimack algorithm). Note: for each H-bond dissociation energy: the 1st cross dash is $P_{i_{max}}$, the 2nd cross dash is the bottom of the range “Maximum”, the 3d cross dash is the top of the range “Minimum”, which was calculated by using non-modified Basov–Jimack algorithm [29]; the red line is OS occurrence frequency under condition when all hydrogen bonds in DNA are ^1^H ($P_{0}$ ).

**Figure 3:**
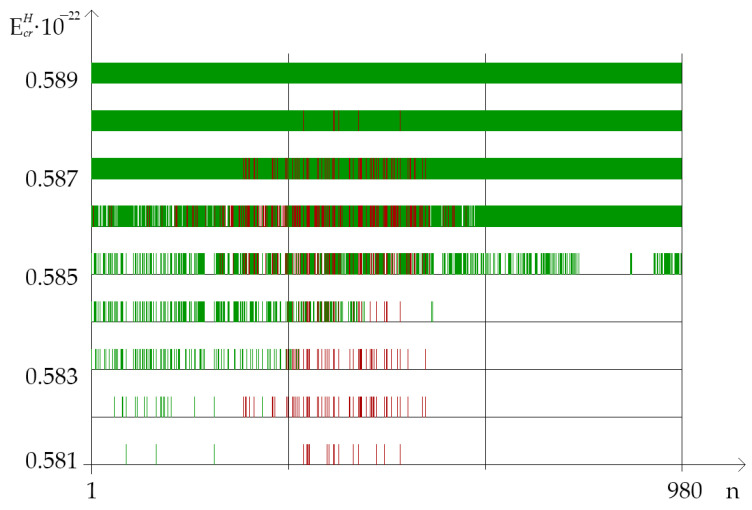
Distribution of nucleobase pairs (which was counted by modified Basov–Jimack algorithm in the different parts of IFNA17), leading after the single ^2^H/^1^H replacement to the extreme frequencies of OS and CSNB occurrences. Note: red dot is the location of the deuterium atom in the DNA molecule, which leads to the maximum probability of OS occurrence (range); green dot is the location of the deuterium atom in the DNA molecule, which leads to the CSNB occurrence (range).

The open state (OS) occurrence frequency was counted by using above-mentioned and modified Basov–Jimack algorithm, according to which, for the highest critical energy range (from $0.581\times 10^{-22}$ N·m to $0.589\times 10^{-22}$ N·m), on Figure 2 the dynamics of the OS occurrence in studied gene dependent on the H-bond dissociation energy under natural condition and after the single ^2^H/^1^H replacement are showed. This data, more than anything else, demonstrates that, in the highest critical energy range, the OS occurrence frequency under natural condition, when all hydrogen bonds in DNA nucleotides are ^1^H, is always lower than frequency of OS occurrence in the range “Maximum”, when is the single replacement of protium with deuterium at nitrogenous bases result in the increase of the OSs ($P_{0}<P_{i}$). Moreover, after $E_{cr}^{H}$ equals to $0.586\times 10^{-22}$ N·m the natural OS occurrence frequency declines abruptly to 0.0 and, further, it has not positive value throughout $E_{cr}^{H}$ diapason from $0.587\times 10^{-22}$ N·m to $0.589\times 10^{-22}$ N·m (Figure 2 and Figure 3). For instance, 22.7% ^2^H-substituted nucleobases in the whole gene increase OS occurrence frequency more than 0.0 under $E_{cr}^{H}$ equals to $0.587\times 10^{-22}$ N·m (Table 1).

On Figure 3, in the different parts of IFNA17, showed the distribution of nucleobase pairs leading to the maximum probability of OS and CSNB occurrences after the single ^2^H/^1^H replacement under energy diapason $\left(E_{cr}^{H}\right)$ from $0.581\times 10^{-22}$ N·m to $0.589\times 10^{-22}$ N·m (henceforth 0.581–0.589).

In our previous study for $0.30–0.58\times 10^{-22}$ N·m energy range [29], the inequality in the frequency of the open states occurrence because of the single ^2^H/^1^H replacement in gene encoding interferon alpha 17 (IFNA17, which consists of 980 nucleotide pairs [30]) was proved through it conditionally divided into three equal parts: from the 1st to the 327th nucleobases (I part), from the 328th to the 653th nucleobases (II part) and from the 654th to the 980th nucleobases (III part). This approach allowed us to contemplate the influence on probabilities ($P_{i}$) of open state occurrences between different nitrogenous bases in double-stranded DNA dependent on the single ^2^H/^1^H replacement in basepair of each gene region. Although these studied parts of IFNA17 had approximately the same number of base pairs, the A-T/G-C ratios were significantly different in each part [31], which are presented in more detail in Table 2.

According to this fact, for the range of the energies from $0.581\times 10^{-22}$ N·m to $0.589\times 10^{-22}$ N·m both quantity of closed states of nitrogenous bases (CSNB) and number of nucletides of the range “Maximum” ($n_{\max}$) for different nitrogenous bases in each of three parts of IFNA17 under single ^2^H/^1^H replacement via BJ-algorithm were calculated, and obtained data are presented in Table 1 for CSNB and Table 3 for $n_{\max}$.

It is worth noting that the change in energy, which was taken into account when calculating CSNB (Table 1) and $n_{max}$ (Table 3) in the highest energy range, always equaled 0.001.

In our study was found out that quantity of CSNB and $n_{\max}$ of OSs for the each gene part were different in the above-described energies $(E_{cr}^{H}$) and depend on A-T/G-C ratio (Table 1 and Table 3). In the range of $E_{cr}^{H}$ from 0.581 to 0.589 the highest total number of $n_{\max}$ counted by the BJ-algorithm was when the single ^2^H/^1^H replacement had taken place in the II part of IFNA17. The number of *i*, which were included in the range “Maximum” in this gene part was in 3.21 times higher than in the I part ($\chi_{B}^{2}$: p<0.001); whereas counted $n_{\max}$ was equal to 0.0 for any of studied energies when ^2^H/^1^H replacing had occurred in the III part (Figure 3). Albeit the A-T nitrogenous bases prevalence over G-C ones in IFNA17 (in 1.48 times), the distribution of the total $n_{\max}$ in the whole gene due to the isotopic substitution at the A-T and G-C nucleotides showed the predominance of ^2^H/^1^H replacement at the cytosine and guanine bases, the number of which was in 3.67 times higher, than the number of ^2^H-substituted A-T ($p\chi_{B}^{2}<0.0001$). Wherein, the difference between $n_{\max}$ of ^2^H/1H-substituted A-T and G-C nucleotides was much more pronounced in the gene I part (in 8.5 times, Table 3) compared to II part (in 3.03 times, Table 3). In addition, the overwhelming majority of ^2^H/^1^H-replacement at the G-C pairs, leading to the $n_{\max}$, arose in the diapason of $E_{cr}^{H}$ from 0.581 to 0.585, so that the number of them was higher in 7.56 times than the nmax via the single ^2^H-substituted adenine or thymine in the same range of energies ($p\chi_{B}^{2}<0.0001$). For $E_{cr}^{H}$ from 0.586 to 0.589, there is a less pronounced difference between deuterium substituted A-T and G-C $n_{\max}$, although the number of the last is more in 2.29 times ($p\chi_{B}^{2}<0.00001$, Table 3).

The calculated quantity of CSNB firstly turned higher in the I part of IFNA17 (under $E_{cr}^{H}$ from 0.581 to 0.582) compared to the II and III parts, which having nothing of these (Figure 3). Further, under $E_{cr}^{H}$ from 0.583 to 0.584 the CSNBs arise in the gene II part; but even at energy equal to 0.584 their number keeps less in 2.75 times than in the I part. Only upon reaching the influence of 0.585 energy in the III part appears some CSNBs, the number of which was less compared to the I and II parts in 1.42 and 1.69 times, respectively ($\chi_{B}^{2}$: p<0.03, Figure 3). However, starting with $E_{cr}^{H}$ equal to 0.586, the quantity of CSNBs in the III part was grown very abruptly, reaching 100% of the nucleotides of this gene part, and it exceeded the CSNB numbers, which were almost equal to each other, in the I and II parts of IFNA17 in 1.51 and 1.56 times, respectively ($\chi_{B}^{2}$: p<0.006, Figure 3). In addition, as in the coming into being of $n_{\max}$, the influence of A-T and the G-C ratio was explicit for generation of CSNBs under the single deuterium substitution in each part of the studied gene. In the 0.581–0.582 range of critical energies, the CSNBs were initiated and grew up specific via the ^2^H-substituted G-C pairs, and, moreover, in the 0.583–0.584 $E_{cr}^{H}$ range the ^2^H-substituted G-C outnumbered the ^2^H-substituted A-T nitrogenous bases and were accounted at least 67% of the total nucleobases leading to the CSNB occurrence in the whole gene ($p\chi_{B}^{2}=0.0056$, Table 1). However, at the $E_{cr}^{H}$ equal to 0.585 this difference of CNBS numbers between ^2^H-substituted A-T and G-C was leveled ($p\chi_{B}^{2}=0.977$). It should be noted, that, under 0.589 $E_{cr}^{H}$, CSNB making was completed for the each nucleobase pairs of the whole IFNA17, and it observed when six G-C pairs had turned into close states in the gene II part specifically ($p\chi_{B}^{2}=0.0108$). In addition, it was found that than the lower A-T/G-C ratio in the gene part, the CSNBs arose there earlier and under lower critical energies. So, for example, in the range of $E_{cr}^{H}$ from 0.581 to 0.585 the Spearman correlation coefficient between A-T/G-C ratio and CSNB numbers was −0.547 (p=0.035, Figure 3). The fractions of the ^2^H-substituted G-C bases in the initiative numbers of CSNB in the each gene parts were following: 100% in the I part under $E_{cr}^{H}$ equal to 0.581 ($p\chi_{B}^{2}=0.2547$), 100% in the II part under $E_{cr}^{H}$ equal to 0.583 ($p\chi_{B}^{2}=0.0359$), and only 30% in the III part under $E_{cr}^{H}$ equal to 0.585 ($p\chi_{B}^{2}=0.771$, Table 1), that could be because of the last gene part had the least number of the G-C nucleobases (Table 2). For the highest acceleration rates of the CSNB numbers, with reaching them approximately half of the total nucleobases in each gene part (but less than all of the nitrogenous bases in the each part) were provided more often due to the ^2^H/^1^H replacement at the A-T pairs: 62% in the I part under $E_{cr}^{H}$ equal to 0.586 ($p\chi_{B}^{2}=0.0068$), 59% in the II part under $E_{cr}^{H}$ equal to 0.586 ($p\chi_{B}^{2}=0.447$) and 70% in the III part under $E_{cr}^{H}$ equal to 0.585 ($p\chi_{B}^{2}=0.771$, Table 1).

## 3. Discussion

The influence of the isotopic ^2^H-effect on nucleic acids structure and their functions is being actively studied, and it is relevant for various fields of science [19,32,33,34,35,36,37,38,39,40]. So, our data allows to understand the mechanism of the possible changes in the OS occurrence frequency in IFNA17 by the single ^2^H/^1^H-replacement under specific energy range more precisely. It is well known about deuterium (D) ability to alter structure and activity of DNA and RNA via changing base-pairing interaction strength by the difference in the energy, stability and geometry between H– and D–bonds, and also through the inequality between ^1^H and ^2^H in the hydrogen bond zero-point vibration energies [32,35,36,41,42,43,44,45,46]. In the present study one of the most vital processes for DNA functioning, which can depend on isotopic ^2^H/^1^H-exchange, was considered—generation of OSs, surplus or deficiency of which can provoke various impairments of DNA stability. It is worth noting that occurrence of nucleotide open states in DNA is very critical conformational transition, which is necessarily needed for both hydrogen exchange and intermolecular interaction of proteins with specific DNA target, which, consequently, provide the realization of the vital function of nucleic acids [47]. In addition, expressing concisely, CSNB are logically inappropriate for the specific implementation of some essential processes in cell due to the formation in DNA many excessively unbreakable sections, which totally prevent the hydrogen exchange between proteins and DNA. Additionally, those energies, under which gene has one or more CSNB (which can equal in summary up to the entirety number of its nucleotides), form the highest critical energy range for the certain gene. Therefore, the study of the influence of ^2^H/^1^H exchange on the molecular dynamic of DNA for energy diapason (or the highest energy range), which initials the rising of the nucleotide closed states in IFNA17, and also taking into consideration A-T/G-C ratio in its different parts, is the great interest for a deeper understanding of the biological functioning of nucleic acids under critical environmental effects [41]. So, according to the data obtained for the studied IFNA17 [29], the highest energy range equals to $E_{cr}^{H}$ from 0.581 to 0.589 for it (Table 1, Figure 3), and that was contemplated in this work.

According to the developed mathematical modeling it was found in the study, that the more increased stability of IFNA17 observed in its parts rich in G-C base pairs, which can be associated with the specific DNA folding of these regions providing more stabilizing energetic contributions and earlier transition to closed states of nucleobases under lower critical energies (starting from the $E_{cr}^{H}$ equals to 0.581 in the gene I part). This statement is confirmed by the significant and negative Spearman correlation coefficient between A-T/G-C ratio and CSNB numbers in the each of the three studied parts of IFNA17. It is well known that even the change of closing nitrogenous bases from G-C to C-G can stabilize DNA hairpin structure approximately 2 kcal/mol and lead to the significant enhance of its melting temperature [48,49]. The similar phenomena are most possible associated with the formation in the alternative DNA structures additional H-bonds, for instance, due to the slightly twisted some nitrogenous base pairs. In addition, it is possible through the occurrence of electrostatically favorable contacts between the partially positively charged amino groups and partially negative oxygen-containing groups with the subsequent formation of different types of loops or other energetically auspicious DNA structures [50,51]. Another but less influenceable reason of the arising CSNB in the gene is increasing of van der Waals packing forces, which changed at the specific conformations of DNA. In opposing to CSNB, the increase OS occurrence frequency, which stems from the presence of the single ^2^H/^1^H-substituted nucleotide in the specific gene region, requires weakening of hydrogen bonds in DNA, reduction of electrostatic attraction between charged chemical groups and decreasing of van der Waals packing forces, that occurred firstly and more often when ^2^H/^1^H replacement takes place at the cytosine and guanine bases, especially in the II part of IFNA17. It can be induced by different reasons: some conformational change of the gene, specific molecular folding, and reducing rate of the rare tautomer formation within DNA because of the isotopic ^2^H/^1^H exchange in nitrogenous bases [52,53]. In our study was showed, that the single ^2^H/^1^H replacement at G-C pairs has the most influence on the initial arise both $n_{\max}$ and $n_{\mathrm{CSNB}}$. Moreover, the last OSs of IFNA17 under $E_{cr}^{H}=0.588\times 10^{-22}$ N·m were presented only G-C pairs (Table 1). The peculiarity of the gene III part with a significant higher content of A-T is the concise turning OSs into CSBNs within two critical energies, such as a collapse (Figure 3). It can point out the higher destabilizing of DNA region enriched A-T nucleobases and risk of its sharp dysfunction under critical conditions [54], nevertheless, the initial OS frequency changes occur foremost because of the ^2^H-substitution at G-C pairs.

In addition, the above modification of the BJ-algorithm was due to the fact that, in the highest critical energy range, there was a progressive accumulation of the number of CSNB (that was starting from $E_{cr}^{H}$ eaqualed 0.581 when for the first time the minimum $P_{i}$ limit was reached: $P_{i}=0.0$). Further, this did not allow the differential consideration of the contribution of the de novo emerging CSNBs (under non-modified BJ-algorithm [29]), which were beginning continually rising from $E_{cr}^{H}$ equaled 0.582 (Figure 3). So that, since the subsequent CSNBs additively accumulated in the total number of the previously formed CSNBs, the role of each emerging CSNB has been indistinguishable in the total number of CSNBs (Figure 3).

Bearing in mind following prerequisites: the contribution of earlier arisen CSNB is clearly higher in failure of the molecular dynamics of the gene, than the later ones, and for the newly formed CSNB it is also inversely proportional to the summary number of the ones in gene, as well as the number of CSNBs are limited by the total number of gene nucleobase pairs the modified BJ-algorithm can required additional below-presented changes for this study:

*i* ϵ range “Minimum” (CSNB): if $0<n_{g_{\mathrm{CSNB}}}/n_{g}\;\leq \;1$:

$n_{\mathrm{CSNB}}=n_{x_{\mathrm{CSNB}}}\left(E_{cr}^{H}\right)\times {\left(1-n_{g_{\mathrm{CSNB}}}\left(E_{cr}^{H}\right)/n_{g}\right)}^{2}$; where:

CSNB is closed state of nitrogenous bases: PCSNB = 0;

$n_{x_{\mathrm{CSNB}}}$ is number of closed states of nitrogenous bases in the gene part *x* under certain $E_{cr}^{H}$;

$n_{g_{\mathrm{CSNB}}}$ is total number of closed states of nitrogenous bases in the whole gene under certain $E_{cr}^{H}$;

$n_{g}$ is number of nitrogenous base pairs in the whole gene;

If $P_{i}\leq P_{i_{min}}+\frac{3}{4}\left(P_{0}-P_{i_{min}}\right)$*: i* ϵ range from Q_2_-min to Q_4_-min (Q_2_–Q_4_-min) [29].

**Figure 4:**
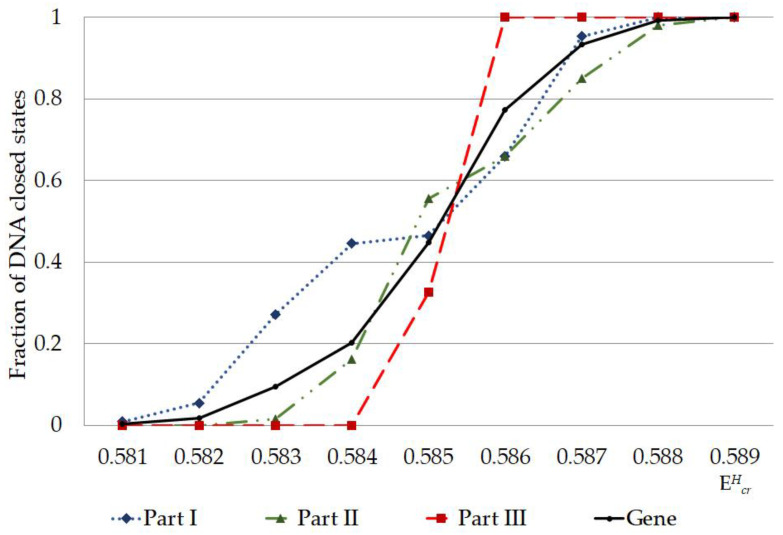
Fraction of nucleobase pairs, which was counted by modified Basov-Jimack algorithm in the different parts of IFNA17, leading after the single ^2^H/^1^H replacement to the CSNB occurrences under the highest critical energy range. Note: fraction of CSNB was calculated as CSNB number under specific $E_{cr}^{H}$ divided by the total nucleobase number in the each part of gene; fraction of CSNB in the gene was calculated as CSNB number in the whole gene under specific $E_{cr}^{H}$ divided by the total nucleobase number in the gene (n = 980); part I is from 1st nucleotide to 327th nucleotide; part II is from 328th nucleotide to 653d nucleotide; part III is from 654th nucleotide to 980th nucleotide.

**Figure 5:**
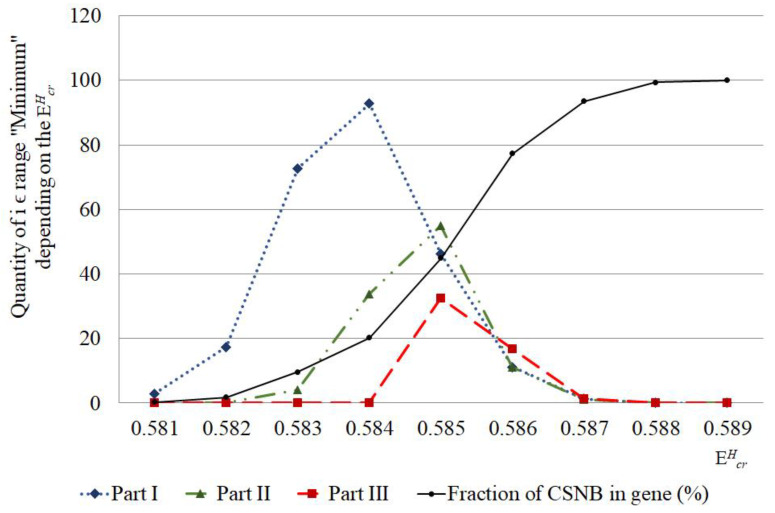
Number of nucleobase pairs, which was counted by the new approach of modified Basov–Jimack algorithm in the different parts of IFNA17, leading after the single ^2^H/^1^H replacement to the CSNB occurrences under the highest critical energy range. Note: fraction of CSNB in the gene was calculated as CSNB number in the whole gene under specific $E_{cr}^{H}$ divided by the total nucleobase number in the gene (n = 980) and then multiplying this ratio by 100%.

The new approach can not only take into account the bigger role earlier formed CSNBs for the certain gene functioning (due to accounting for $n_{x_{\mathrm{CSNB}}}\left(E_{cr}^{H}\right)$ depending on $n_{g_{\mathrm{CSNB}}}\left(E_{cr}^{H}\right)/n_{g}$, but also avoid the cumulative effect distorting the total CSNB number (especially for the higher energies: $E_{cr}^{H}\geq 0.586$, Figure 3 and Figure 4) by using square function for the component: $1-n_{g_{\mathrm{CSNB}}}\left(E_{cr}^{H}\right)/n_{g}$. Foremost this approach allows us to reduce the number of false positive data when evaluating CSNBs (Figure 3 and Figure 5). Other benefits of the changes in the new approach of BJ-algorithm and some disadvantages of decile and quartile methods for selection of the nucleobases for the maximum and minimum ranges were presented in details in our earlier work [29].

**Figure 6:**
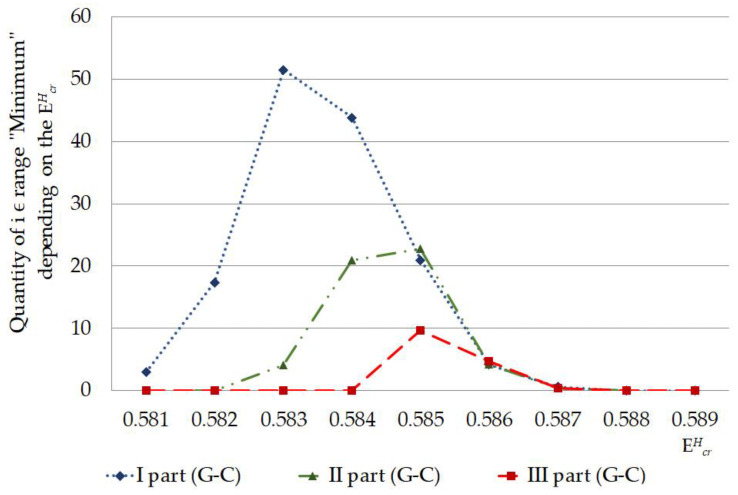
Quantity of nucleobase pairs, which was counted by the new approach of modified Basov–Jimack algorithm in the different parts of IFNA17, leading after the single ^2^H/^1^H-substitution at G-C bases to the CSNB occurrences under the whole highest critical energy range.

For instance, if we confront statistics of the data, processed by non-modified BJ-algorithm and the new approach, it can be found when comparing their results, there is no significant difference between the sums of $n_{\mathrm{CSNB}}$, which were counted by the non-modified BJ-algorithm [29], in the second (II) and third (III) parts of the gene throughout 0.581–0.589 energy range (*n_(part-II_*_)_ = 1371 bases, *n_(part-III_*_)_ = 1415 bases, $p\chi_{Yates}^{2}=0.3927$), but there is significant difference on 205.9% between the sums of $n_{\mathrm{CSNB}}$, higher in the II part compared to III part, are calculated by the new approach in the same energy diapason (*n_(part-II_*_)_ = 105 bases, *n_(part-III_*_)_ = 51 bases, $p\chi_{Yates}^{2}<0.0001$). It was reached due to the strong reduction of the false positive results because of the above-mentioned new approach of the modified BJ-algorithm (Figure 3 and Figure 5). Additionally, when CSNB numbers were counted by the new approach of the modified BJ-algorithm in the whole gene and for each $E_{cr}^{H}$ throughout the energy diapason from 0.581 to 0.588, the strong correlation between the total $n_{\mathrm{CSNB}}$ was revealed (because of ^2^H-substitution at both A-T and G-C) and $n_{\mathrm{CSNB}}$ due to the ^2^H-substitution only at G-C nucleobases, with Spearman’s coefficient equaled to 0.881 (p=0.00385), which proved the far higher ability of ^2^H-substituted G-C produced more evenly and incessantly the closed states in IFNA17 compared to ^2^H-substituted A-T bases (Figure 6).

**Figure 7:**
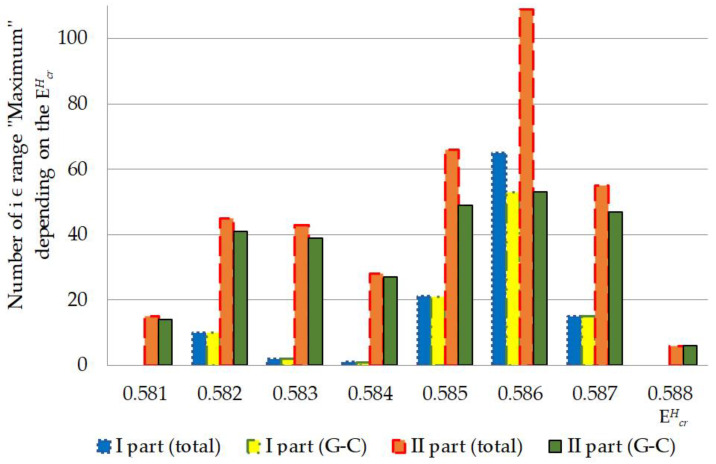
Numbers of nucleobases from the range “Maximum”, which were counted by the modified Basov–Jimack algorithm, in the I and II parts of IFNA17 throughout 0.581–0.588 energy diapason. Note: Total $n_{max}$ in each gene part was calculated as $n_{max}$ number due to the single ^2^H/^1^H-exchange both at A-T and G-C pairs in total; G-C $n_{max}$ in each gene part was calculated as $n_{max}$ number due to the single ^2^H/^1^H-exchange only at G-C pairs.

In general, the developed method of the mathematical modeling provide unprecedented insight into the DNA behavior under the highest critical energy range, which greatly expands scientific understanding of nucleobases interaction [50]. Under the highest critical energy range, these described fluctuations of $n_{\mathrm{CSNB}}$ after single ^2^H/^1^H-substitutions in the different gene parts can provoke DNA dysfunction, for example, because of the sharp slowdown in the rate of the generation of vital non-superhelical denaturation bubbles, which have more than four nucleobase-pairs in the promoter regions and specific binding points for DNA repair proteins [55,56] that can be a predictor of DNA repair system lesion. In addition, obtained data demonstrate the possibility of higher risk, which increase abruptly in the whole energy range from 0.581 to 0.587 of altering and impairment of IFNA17 due to the single ^2^H/^1^H exchange in the A-T richest part compared to the both I or II parts, especially under $E_{cr}^{H}$ more than 0.585 (Figure 3 and Figure 4). It should be noted, that in opposition to the natural frequency of OSs in the gene under the energy higher 0.586, after the single ^2^H-substitution at G-C in the II part observed lower $n_{\mathrm{CSNB}}$, so this can provide more probability of DNA bubble occurrence, be one of nucleic acid adaptation mechanisms to critical conditions, and matter to the evolution process of living system [57]. According to the developed mathematical modeling and modified BJ-algorithm it was found, that, under the $E_{cr}^{H}$ from 0.586 to 0.588 the occurrence $P_{i}$ rates, which matches the range “Maximum”, due to the ^2^H/^1^H exchange at G-C is always significant more than ones due to the same isotopic exchange at A-T, that can consider as the positive participating of the ^2^H-substituted guanine and cytosine in nucleic acid adaptation in opposing to the ^2^H-substituted adenine and thymine, which give no advantages to DNA denaturation process. For example, it can influence the rate of specific DNA bubble forming, which participating in generation of the pre-existing state of denaturation, that is required by specific DNA-binding enzymes [58,59]. The relationship study between total $n_{max}$ and $n_{max}$ produced due to the ^2^H/^1^H substitution at G-C in the I and II parts of IFNA17, which had more than one ^2^H-substituted bases from range “Maximum” in opposing to III part, for each $E_{cr}^{H}$ throughout the energy diapason from 0.581 to 0.588 revealed Spearman’s coefficient equals to 0.994 (p<0.00001), that pointed out the obvious dominant role of ^2^H-substituted G-C bases in the generation $n_{max}$ in these gene parts compared to A-T ones (Figure 7). Moreover, the study demonstrated, that the single deuterium introduction in the gene III part, which is out of the coding region from 50th to 619th base pairs [60,61], had the most close data of the OS occurrence rate compared to the data of OS occurrence frequency under condition when all hydrogen bonds in IFNA17 are ^1^H, that was showed clearly the least possibility of ^2^H/^1^H replacement in this part to influence on gene adaptation process under critical conditions positively.

Above-described cases of the double-stranded DNA dynamics obviously confirmed that under the highest critical energy diapason, the heterogeneous sensitivity of IFNA17 to isotopic ^2^H/^1^H exchange took place and frequency of OS occurrence strongly depended on not only the single ^2^H-modified nucleobase in the certain gene region (I, II, or III) but, additionally, on the A-T/G-C-ratio in each of its part; the last was, especially, correlated to the quantity of initial CSNB, that was proved due to significant and negative Spearman’s coefficient, reflecting the moderate strength of relationship between them under energy range from 0.581 to 0.585.

So, below are the main inferences of our work, confirming in detail the above-mentioned conclusion:

1. In some cases, the single ^2^H/^1^H replacement result in positive value of OS occurrence frequency in the IFNA17 throughout $E_{cr}^{H}$ diapason from $0.586\times 10^{-22}$ N·m to $0.588\times 10^{-22}$ N·m opposing to the OS occurrence frequency, when all hydrogen bonds in DNA nucleotides are ^1^H, which is always equaled to 0.0 after $E_{cr}^{H}$ exceed $0.585\times 10^{-22}$ N·m. This underlines that at least 22.7% of the total number ^2^H-substituted nucleobases can reduce molecular interaction in the studied gene and increase the hydrogen bond dissociation, foremost in its part from 328 to 653 nitrogenous bases;
2. The counted occurrences of the OSs were much higher when the single ^2^H/^1^H replacement had taken place at nucleobases of the middle part IFNA17 (from 328 to 653 nucleotides) compared to its other parts, and this had a strong prevalence rate in the G-C pairs;
3. The lowest rate of the OS occurrence was under the single deuterium substitution at the nitrogenous bases in the gene III part (from 654 to 980 nucleotides), which was also too rich in A-T pairs (72.2%) compared to the other parts of IFNA17, so that the calculated $n_{max}$ was equal to 0.0 for all of the studied critical energies (from $0.581\times 10^{-22}$ N·m to $0.589\times 10^{-22}$ N·m);
4. Sum of $n_{max}$ was less significant when the single ^2^H/^1^H replacement occurred at the A-T nucleobase pairs compared to the G-C ones in the I and II parts of IFNA17, and the relationship between total $n_{max}$ and $n_{max}$ at G-C in these parts for each $E_{cr}^{H}$ throughout the energy diapason from $0.581\times 10^{-22}$ N·m to $0.588\times 10^{-22}$ N·m was strong ($r_{\mathrm{Spearman}}=0.994$) that proves the obvious dominant role of ^2^H-substitutied G-C bases in the generation $n_{max}$ compared to the A-T;
5. Earliest CSNBs (n = 3) arose under $E_{cr}^{H}$ equal to $0.581\times 10^{-22}$ N·m when IFNA17 had had the single ^2^H-substituted cytosine or guanine nitrogenous bases in its I part (from 1 to 327 nucleotides). Moreover, throughout the range energy from $0.581\times 10^{-22}$ N·m to $0.583\times 10^{-22}$ N·m, the single ^2^H/^1^H replacement, leading to the CSNBs, prevailed in the I part, especially for its G-C pairs making up at least 67% of the total CSNB quantity in the whole gene. So, for IFNA17 throughout the range of $E_{cr}^{H}$ from $0.581\times 10^{-22}$ N·m to $0.585\times 10^{-22}$ N·m the Spearman correlation coefficient between A-T/G-C ratio in the each gene part and CSNB numbers was significant and negative *(*$r_{\mathrm{Spearman}}=-0.547$);
6. The highest acceleration of CSNB occurrence was observed when the single ^2^H/^1^H replacement took place at nucleotides of the III part of IFNA17 under $E_{cr}^{H}$ from $0.585\times 10^{-22}$ N·m to $0.586\times 10^{-22}$ N·m, and throughout it they very abruptly reached the value of 100% of the nucleobases in this gene part. It indicates the obvious and higher vulnerability of IFNA17 due to the single ^2^H-substitution at nucleobases from 654 to 980 compared with other gene parts exposed to studied critical energies, which increased the risk of permanent disorders of converting genetic information to mRNA messenger;
7. All of the above-mentioned underline clearly the significant difference in the responsiveness of each IFNA17 parts under range of critical energies because of the single ^2^H/^1^H replacement in their nucleobases and with its strong dependence on A-T/G-C ratio with the prevalent contribution of the last pair, which leads to an increase due to the ^2^H-substitution into its nitrogenous bases both $n_{max}$ and CSNB, especially under $E_{cr}^{H}$ diapason from $0.581\times 10^{-22}$ N·m to $0.584\times 10^{-22}$ N·m;
8. The single ^2^H-substitution at G-C pairs not only had the most influence on the initial arise both $n_{max}$ and $n_{\mathrm{CSNB}}$ in the whole gene throughout the critical energy diapason from $0.581\times 10^{-22}$ N·m to $0.584\times 10^{-22}$ N·m but also made possible the existence of the last OS occurrence under $E_{cr}^{H}$ equals to $0.588\times 10^{-22}$ N·at least in six cases that proves the leading effect of the isotopic ^2^H/^1^H modifications at G-C compared to A-T on the molecular dynamics of IFNA17;
9. In addition, in the study was presented a modified algorithm allowing for accounting for nucleobases with the single ^2^H/^1^H replacement, which leads to occurrence of both the highest rate of Oss and CSNBs. Also, it showed the developed approach, decreasing significantly the false positive results compared to non-modified BJ-algorithm [29] due to the differentiated counting of the total sum of CSNB occurrence in the gene with relevance to the critical energy in the highest diapason.

## 4. Materials and Methods

### 4.1. Mathematical Model

The open states of the DNA molecule and its dynamics are well described by a mechanical model, which is two chains of disks that are interconnected by transverse springs; this system is described by the following Newton equations:

(1) $$I_{1}^{i}\frac{d^{2}\varphi_{1}^{i}\left(t\right)}{dt^{2}}=K_{1}^{i}\left[\varphi_{1}^{i-1}\left(t\right)-2\varphi_{1}^{i}\left(t\right)+\varphi_{1}^{i+1}\left(t\right)\right]-\;\delta^{i}\left(k_{12}^{i}R_{1}^{i}(R_{1}^{i}+R_{2}^{i})\sin\varphi_{1}^{i}+k_{12}^{i}R_{1}^{i}R_{2}^{i}\sin(\varphi_{1}^{i}-\varphi_{2}^{i})\right)+\;F_{1}^{i}\left(t\right),i=\bar{2,n-1},$$

(2) $$I_{1}^{1}\frac{d^{2}\varphi_{1}^{1}\left(t\right)}{dt^{2}}=K_{1}^{1}\left[\varphi_{1}^{2}\left(t\right)-\varphi_{1}^{1}\left(t\right)\right]-\;\delta^{i}\left(k_{12}^{1}R_{1}^{1}(R_{1}^{1}+R_{2}^{1})\sin\varphi_{1}^{1}+k_{12}^{1}R_{1}^{1}R_{2}^{i}\sin(\varphi_{1}^{1}-\varphi_{2}^{1})\right)+F_{1}^{1}\left(t\right),$$

(3) $$I_{1}^{n}\frac{d^{2}\varphi_{1}^{n}\left(t\right)}{dt^{2}}=K_{1}^{n}\left[\varphi_{1}^{n-1}\left(t\right)-\varphi_{1}^{n}\left(t\right)\right]-\;\delta^{i}\left(k_{12}^{n}R_{1}^{n}\left(R_{1}^{n}+R_{2}^{n}\right)\sin\varphi_{1}^{n}+k_{12}^{n}R_{1}^{n}R_{2}^{n}\sin\left(\varphi_{1}^{n}-\varphi_{2}^{n}\right)\right)+F_{1}^{n}\left(t\right),$$

(4) $$I_{2}^{i}\frac{d^{2}\varphi_{2}^{i}\left(t\right)}{dt^{2}}=K_{2}^{i}\left[\varphi_{2}^{i-1}\left(t\right)-2\varphi_{2}^{i}\left(t\right)+\varphi_{2}^{i+1}\left(t\right)\right]+\;\delta^{i}\left(k_{12}^{i}R_{2}^{i}(R_{1}^{i}+R_{2}^{i})\sin\varphi_{2}^{i}-k_{12}^{i}R_{1}^{i}R_{2}^{i}\sin(\varphi_{2}^{i}-\varphi_{1}^{i})\right)+\;F_{2}^{i}\left(t\right),i=\bar{2,n-1},$$

(5) $$I_{2}^{1}\frac{d^{2}\varphi_{2}^{1}\left(t\right)}{dt^{2}}=K_{2}^{1}\left[\varphi_{2}^{2}\left(t\right)-\varphi_{2}^{1}\left(t\right)\right]+\;\delta^{i}\left(k_{12}^{1}R_{2}^{1}\left(R_{1}^{1}+R_{2}^{1}\right)\sin\varphi_{2}^{1}1-k_{12}^{1}R_{1}^{1}R_{2}^{1}\sin\left(\varphi_{2}^{1}-\varphi_{1}^{1}\right)\right)+F_{2}^{1}\left(t\right),$$

(6) $$I_{2}^{n}\frac{d^{2}\varphi_{2}^{n}\left(t\right)}{dt^{2}}=K_{2}^{n}\left[\varphi_{2}^{n-1}\left(t\right)-\varphi_{2}^{n}\left(t\right)\right]+\;\delta^{i}\left(k_{12}^{n}R_{2}^{n}\left(R_{1}^{n}+R_{2}^{n}\right)\sin\varphi_{2}^{n}-k_{12}^{n}R_{1}^{n}R_{2}^{n}\sin\left(\varphi_{2}^{n}-\varphi_{1}^{n}\right)\right)+F_{2}^{n}\left(t\right).$$

here:

$\varphi_{j}^{i}\left(t\right)$—is the angular deflection of the *i*-th nitrogenous base of the *j*-th chain counted counterclockwise at time *t*;

$I_{j}^{i}$—is the rotational inertia of the *i*-th nitrogenous base of the *j*-th chain;

$R_{j}^{i}$—is the distance between the center of inertia of the *i*-th nitrogenous base of the *j*-th chain to sugar phosphate chain;

$K_{j}^{i}$—is the constant characterizing the torsion moment of the *i*-th segment of the *j*-th sugar phosphate chain;

$k_{12}^{i}$—is the constant characterizing the bond elastic properties of the *i*-th nitrogenous base pairs;

$F_{j}^{i}\left(t\right)$—external influence on the *i*-th nitrogenous base of the *j*-th chain at a time *t*;

n—is the number of nitrogenous base pairs in the system.

The magnitude of the external impact is assumed to be equal to $F_{j}^{i}\left(t\right)=-\beta_{j}^{i}\frac{d\varphi_{j}^{i}}{dt}\left(t\right)++F_{0}\cos\omega t$, where the term is $-\beta_{j}^{i}\frac{d\varphi_{j}^{i}}{dt}\left(t\right)$ simulates the effects of dissipation caused by interaction with the water surrounding the DNA molecule, the term $F_{0}\cos\omega t$ is an external periodic effect.

In Equations (1)–(6), the first term to the right of the equality sign describes the force acting on the *i*-th nitrogenous base from the sugar-phosphate filament, the second term—the force from the complementary nitrogenous base, the third term—external impact.

Thus, Equations (1)–(6) allow us to model the hydrogen bond in the *i*-th pair ($\delta^{i}=1$, $k_{12}^{i}=k_{12}^{H,i}$), deuterium ($\delta^{i}=1$, $k_{12}^{i}=k_{12}^{D,i}$) and the break of this connection ($\delta^{i}=0$). We will assume that a break in base pairs occurs if the potential binding energy in these pairs exceeds a certain critical value $E_{cr}^{H}$ for a hydrogen bond and $E_{cr}^{D}$ for a deuterium bond, if the potential energy in a pair with a broken bond is less than the critical value, then the bond is restored.

To Equations (1)–(6) we add the initial conditions:

(7) $$\varphi_{1}^{i}\left(0\right)=\varphi_{1,0}^{i},\;\frac{d\varphi_{1}^{i}}{dt}\left(0\right)=\varphi_{1,1}^{i},$$

(8) $$\varphi_{2}^{i}\left(0\right)=\varphi_{2,0}^{i},\;\frac{d\varphi_{2}^{i}}{dt}\left(0\right)=\varphi_{2,1}^{i},\;i=\bar{1,n}.$$

For the sake of certainty, we assume that at t=0 the system is in equilibrium, that is, in the initial conditions (7) and (8)

$$\varphi_{1,0}^{i}=\varphi_{1,1}^{i}=\varphi_{2,1}^{i}=0,\;\;\;\varphi_{2,0}^{i}=\pi ,i=\bar{1,n}.$$

Problems (1)–(8) is a Cauchy problem for a system of 2n ordinary differential equations; in this paper, all studies were carried out on the basis of a numerical solution of this system.

The study of the effect of ^2^H/^1^H exchange on the formation and dynamics of open states will be carried out using the example of the gene encoding interferon alpha 17. For this gene, n=980, the values of the coefficients of Equations (1)–(6) are taken from [31]), the values of $F_{0}=0.526\times 10^{-22}\;J,\;ɷ=0.4\times 10^{12}\;s^{-1}$.

We assume that $E_{cr}^{D}=k^{D}\cdot E_{cr}^{H}$, $k_{12}^{D,i}=k^{D}\cdot k_{12}^{H,i}$ if one of the hydrogen bonds in the *i*-th base pair replaced with deuterium, $k^{D}=1.05$. The value of the $k^{D}$ coefficient will be chosen because the deuterium bond is 5% stronger than the hydrogen bond. The order of the critical energy $E_{cr}^{H}$ is consistent with the experimental data from the work.

We designate by *P*_0_ the probability of an OS formation in a molecule in which all pairs of nitrogenous bases are connected by hydrogen bonds; by *P_i_*, *i* =$\bar{\;1,n}$, the probability of an OS occurrence in a DNA molecule in which the *i*-th nitrogenous base pair one <any> hydrogen bond is replaced by deuterium.

The probabilities *P*_0_ и *P_i_*, *i* = $\bar{1,n}$, will be sought on the basis of a numerical solution of the problem (1)–(8). To do this, we will create a set of points *t_j_* = *jƮ*, *j* =$\;\bar{1,m}$, *Ʈ* = *T/m* in the segment [0, *T*]. Calculate at *t* = *t_j_* the ratio *q_j_* of the number of base pairs with a broken bond to the total number of base pairs *n*, then the value of *P_k_* is equal to the arithmetic mean value over the points *t_j_* of these ratio:

$$P_{i}=m^{-1}\left(\overset{m}{\underset{j=1}{\sum }}q_{j}\right)$$

### 4.2. Modification of Basov–Jimack Algorithm

The affiliation of each nucleotide to I part, II part or III part of IFNA17 was determined due to its sequence (serial) number. Nucleobases with $P_{i_{max}}$, and the higher $P_{i}$ were selected for the maximum range and their sum was $n_{max}$, and nucleobases, which had CSNB (closed state of nitrogenous bases) ($P_{i}=0$), were selected for the minimum range, and their number was designated as $n_{\mathrm{CSNB}}$. According to the below-presented modified Basov–Jimack algorithm (BJ-algorithm [30]), which was changed for the highest critical energy range, every $P_{i}$ was arranged from CSNB to $P_{i_{max}}$ and their numbers were calculated in each gene part:

(1) *i* ϵ range “Maximum” (BJ-max):
  - if $P_{i_{max}}-\frac{1}{10}\left(P_{i_{max}}-P_{i_{min}}\right)\geq P_{0}+\frac{1}{2}\left(P_{i_{max}}-P_{0}\right)$ and $P_{i_{max}}>P_{0}\geq P_{i_{min}}\geq 0$*:*
    $P_{i}\geq P_{i_{max}}-\frac{1}{10}\left(P_{i_{max}}-P_{i_{min}}\right)\Rightarrow n_{max}=\sum nP_{i}$*;* or else:
  - if $P_{i_{max}}-\frac{1}{10}\left(P_{i_{max}}-P_{i_{min}}\right)<P_{0}+\frac{1}{2}\left(P_{i_{max}}-P_{0}\right)$ and $P_{i_{\max}}>P_{0}\geq P_{i_{\min}}\geq 0$:
    $P_{i}\geq P_{i_{max}}-\frac{1}{4}\left(P_{i_{max}}-P_{0}\right)\Rightarrow n_{max}=\sum nP_{i}$*;*
(2) *i* ϵ range “Minimum” (CSNB):
  if $0<n_{g_{\mathrm{CSNB}}}/n_{g}\;\leq \;1:\;n_{\mathrm{CSNB}}=n_{x_{\mathrm{CSNB}}}\left(E_{cr}^{H}\right)$;

where $n_{max}$ is number of *i*, which were included in the range “Maximum”; CSNB: $P_{\mathrm{CSNB}}=0$; $E_{cr}^{H}$ is critical energy in the diapason, which has 1 or more CSNB: $0<n_{g_{\mathrm{CSNB}}}/n_{g}\;\leq \;1$, in this case from $0.581\times 10^{-22}$ N·m to $0.589\times 10^{-22}$ N·m; $n_{x}$ is number of nitrogenous base pairs in *x* part of gene: *x* can be I, II or III part of IFNA17; $n_{x_{\mathrm{CSNB}}}$ is number of closed states of nitrogenous bases in the gene part *x* under specific $E_{cr}^{H}$; $n_{g}$ is number of nitrogenous base pairs in the whole gene.

### 4.3. Statistics

In addition, some statistical methods were used to exact the significance of differences among $n_{\mathrm{CSNB}}$ and $n_{max}$ of the three gene parts. Yates corrected chi-squared test ($\chi_{Yates}^{2}$) was applied for a 2 × 2 contingency table (where degrees of freedom $\left(\nu \right)=\left(2-1\right)\cdot \left(2-1\right)=1)$. As a short-cut, for a 2 × 2 table with the following entries (Table 4):

Where **A** and **B** are the rows according to the parts of the gene: I, II, or III; **S** is the column of $n_{\mathrm{CSNB}}$ of the range “Minimum”, or $n_{max}$ of the range “Maximum”; **F** is the column of the rest number of nucleotide pairs; a and c are $n_{\mathrm{CSNB}}$ of the range “Minimum”, or $n_{max}$ of the range “Maximum” in the each compared part of the gene in the determined range of $E_{cr}^{H}$; b and d equals the total number of nucleotide pairs of the gene part minus $n_{\mathrm{CSNB}}$ of the range “Minimum”, or $n_{max}$ of the range “Maximum” in the each compared part of the gene in the determined range of $E_{cr}^{H}$; $N_{A}=a+b;\;N_{B}=c+d;\;N_{S}=a+c;\;N_{F}=b+d;\;N=N_{A}+N_{B}+N_{S}+N_{F}.$

$$\chi_{Yates}^{2}=N{\left(\left|ad-bc\right|-N/2\right)}^{2}/N_{A}\cdot \;N_{B}\cdot N_{S}\cdot N_{F}.$$

Chi-square corrected by procedure for Bonferroni ($\chi_{B}^{2}$) was used for a 3 × 2 contingency table (where 3 is rows, 2 is columns, $\nu =\left(3-1\right)\cdot \left(2-1\right)=2)$. The Kruskal–Wallis ANOVA by Ranks test (KWt) was applied for the comparison $n_{\mathrm{CSNB}}$ of the range “Minimum”, or $n_{max}$ of the range “Maximum” in the determined range of $E_{cr}^{H}$ for two gene parts are mutually independent. The assess of the relationship between two variables was measured by Spearman’s rank correlation coefficient ($r_{\mathrm{Spearman}}$).

## 5. Conclusions

Thus, in the study, it was found that under the highest critical energy range, the very significant inequality of the frequencies of OS occurrence in the full gene were due to the single ^2^H-substitution at the nucleobase of its different regions, which were almost equal to each other (gene I, II, III parts) by the number of nucleotides. In almost the entire highest critical energy range after the single ^2^H/^1^H replacement at the nitrogenous base of IFNA17, the $P_{i}$ values both in the range “Maximum” and “Minimum” were much different compared to the OS occurrence frequency under condition when all hydrogen bonds in DNA are ^1^H ($P_{0}$, Figure 2). Foremost, the A-T/G-C ratio had a strong influence on the frequency of CSNB in the $E_{cr}^{H}$ diapason from $0.581\times 10^{-22}$ N·m to $0.588\times 10^{-22}$ N·m that can be not only the reason of the function impairment of IFNA17 but can also demonstrate the nucleic acid adaptation mechanisms to critical conditions and matter to the evolution process of living system [57]. For instance, under the highest critical energy range, gene function impairment can occur because of the increase in $n_{\mathrm{CSNB}}$, leading to the sharp slowdown of DNA bubble generation, which has more than four nucleobases, participating in forming of the preexisting denaturation state that is required by specific DNA-binding proteins [58,59]. In turn, via the single ^2^H/^1^H replacement at certain bases, the $n_{max}$ arising can be the mechanism of preserving the vital functioning of IFNA17 under critical conditions. The modified algorithm and new approach allow for calculating the $n_{max}$ and $n_{\mathrm{CSNB}}$ throughout the highest critical energy diapason, in opposition to the non-modified Basov–Jimack algorithm, which is appropriate for counting the base-pair number in the range “Maximum” and “Minimum” under only the critical energies less than the highest range.

In addition, it should be noted, that the developed mathematical modeling showed its appropriateness for counting of the OS occurrence under the whole highest critical energy range. However, our study had a limitation, which was following: all results had been obtained in the frameworks of the mathematical model based on Newton’s equations and are representative of the Cauchy problem for the system of 2n ordinary differential equations, which did not entirely take into account the all contribution of DNA–protein interactions [62,63]. Nevertheless, we do not contemplate this limitation as an insurmountable obstacle and consider the modified algorithm and new approach as appropriate methods that can be generalized and used for more other complex models of DNA dynamics.

## Figures and Tables

**Table 1:** Quantity of closed states of nitrogenous bases for different nitrogenous bases in each of three parts of IFNA17 dependent on single ^2^H/^1^H replacement.

$E_{cr}^{H}\cdot 10^{-22},\;N\cdot m$	$P_{0}\cdot 10^{-5}$	Part of Gene	A–T (CSNB, *n*)	G–C (CSNB, *n*)
0.581	1.02	I	0	3
		II	0	0
		III	0	0
0.582		I	0	18
	0.99	II	0	0
		III	0	0
0.583	0.96	I	26	63
		II	0	5
		III	0	0
0.584		I	77	69
	0.93	II	20	33
		III	0	0
0.585	0.84	I	77	69
		II	106	75
		III	75	32
0.586	0	I	134	82
		II	127	82
		III	236	91
0.587	0	I	163	149
		II	178	93
		III	236	91
0.588	0	I	163	164
		II	186	134
		III	236	91
0.589	0	I	163	164
		II	186	140
		III	236	91

Note: CSNB is closed states of nitrogenous bases (PCS = 0), A—adenine, T—thymine, G—guanine, C—cytosine.

**Table 2:** Distribution of adenine-thymine (A–T) and guanine-cytosine (G–C) base pairs in different parts of IFNA17.

Part of IFNA17	Nucleobase Pair Quantity	A–T Ratio (%)	G–C Ratio (%)
I	327 (from 1 to 327)	49.8	50.2
II	326 (from 328 to 653)	57.1	42.9
III	327 (from 654 to 980)	72.2	27.8

**Table 3:** Quantity of A-T and G-C nucleobases, included in the range “Maximum”, in each of three parts of IFNA17 dependent on single ^2^H/^1^H replacement.

$E_{cr}^{H}\cdot 10^{-22},\;N\cdot m$	$P_{0}\cdot 10^{-5}$	$P_{i_{max}}\cdot 10^{-5}$	Part of Gene	$A–T\;(n_{max},\;n)$	$G–C\;(n_{max}B,\;n)$
0.581	1.02	1.09	I	0	0
			II	1	14
			III	0	0
0.582	0.99	1.07	I	0	10
			II	4	41
			III	0	0
0.583	0.96	1.05	I	0	2
			II	4	39
			III	0	0
0.584	0.93	1.03	I	0	1
			II	1	27
			III	0	0
0.585	0.84	1	I	0	21
			II	17	49
			III	0	0
0.586	0	0.97	I	12	53
			II	56	53
			III	0	0
0.587	0	0.94	I	0	15
			II	8	47
			III	0	0
0.588	0	0.89	I	0	0
			II	0	6
			III	0	0
0.589	0	0	I	0	0
			II	0	0
			III	0	0

Note: A—adenine, T—thymine, G—guanine, C—cytosine.

**Table 4:** Chi-squared test with Yates correction.

	S	F	
** A **	* a *	* b *	$N_{A}$
** B **	* c *	* d *	$N_{B}$
	$N_{S}$	$N_{F}$	N

## Data Availability

Not applicable.
